# Specific and sensitive detection tools for *Xanthomonas arboricola* pv. corylina, the causal agent of bacterial blight of hazelnut, developed with comparative genomics

**DOI:** 10.3389/fpls.2023.1254107

**Published:** 2023-09-13

**Authors:** Monika Kałużna, Andjelka Prokić, Aleksa Obradović, William A. Weldon, Virginia O. Stockwell, Joël F. Pothier

**Affiliations:** ^1^ The National Institute of Horticultural Research, Skierniewice, Poland; ^2^ University of Belgrade, Faculty of Agriculture, Belgrade, Serbia; ^3^ Valent BioSciences, Libertyville, IL, United States; ^4^ United States Department of Agriculture, Agricultural Research Service, Horticultural Crops Disease and Pest Management Research Unit, Corvallis, OR, United States; ^5^ Environmental Genomics and Systems Biology Research Group, Institute for Natural Resource Sciences, Zurich University of Applied Sciences (ZHAW), Wädenswil, Switzerland

**Keywords:** *Corylus* spp., *Corylus avellana*, diagnosis, PCR, LAMP, qPCR

## Abstract

*Xanthomonas arboricola* pv. corylina (*Xac*; formerly *Xanthomonas campestris* pv. corylina) is the causal agent of the bacterial blight of hazelnuts, a devastating disease of trees in plant nurseries and young orchards. Currently, there are no PCR assays to distinguish *Xac* from all other pathovars of *X*. *arboricola*. A comparative genomics approach with publicly available genomes of *Xac* was used to identify unique sequences, conserved across the genomes of the pathogen. We identified a 2,440 bp genomic region that was unique to *Xac* and designed identification and detection systems for conventional PCR, qPCR (SYBR^®^ Green and TaqMan™), and loop-mediated isothermal amplification (LAMP). All PCR assays performed on genomic DNA isolated from eight *X*. *arboricola* pathovars and closely related bacterial species confirmed the specificity of designed primers. These new multi-platform molecular diagnostic tools may be used by plant clinics and researchers to detect and identify *Xac* in pure cultures and hazelnut tissues rapidly and accurately.

## 1 Introduction

*Xanthomonas arboricola* pv. corylina (*Xac*; formerly *Xanthomonas campestris* pv. corylina; Vauterin et al., 1995) is a Gram-negative plant pathogenic bacterium of the *Lysobacteraceae* family (earlier synonym of *Xanthomonadaceae*) (Saddler and Bradbury, 2005; Tindall, 2014). *Xac* is the causal agent of the bacterial blight of hazelnut (*Corylus avellana* L.). Other *Corylus* spp., including *C. pontica*, *C. maxima* and *C. colurna*, also can be infected by *Xac* (OEPP/EPPO, 1986; OEPP/EPPO, 2004). Bacterial blight of hazelnut is a devastating disease that is commonly observed in plant nurseries and young orchards, causing significant plant mortality (Miller et al., 1949; Moore, 2002; OEPP/EPPO, 2004; Lamichhane and Varvaro, 2014; Webber et al., 2021). The disease also can be seen in established production orchards, especially on susceptible cultivars. The main disease symptoms include angular necrotic lesions on leaves and the involucres of shells, as well as shoot necrosis and cankers. Lesions on the stalk and top of nuts results in reduced nut quality. Dieback of nut-bearing branches causes measurable yield reduction. Over time, bacterial blight of hazelnut reduces tree health and results in poor tree structure and continued yield losses (Obradović et al., 2010; Kałużna et al., 2021).

*Xac* has been a regulated pathogen and placed on the European and Mediterranean Plant Protection Organization (EPPO) list A2 of quarantine pathogens, but it was recently reclassified as a Regulated Non-Quarantine Pest (RNQP) (European Union, 2016; European Union, 2019). Currently, bacterial blight caused by *Xac* has been reported in nearly every hazelnut-producing country (OEPP/EPPO, 2004; Kałużna et al., 2021; Osdaghi, 2022). Identification of *Xac* is currently a tedious, multistep process, which is described below and can take several days to return a diagnostic result. Difficulty in identification arises largely because it is closely related to seven other pathovars of *X. arboricola*, including pv. pruni (*Xap*), pv. juglandis (*Xaj*), pv. fragariae, pv. celebensis, pv. arracaciae, pv. poinsettiicola and pv. zantedeschiae (Vauterin et al., 1995; Janse et al., 2001; Fischer-Le Saux et al., 2015; Kałużna et al., 2021). Two former *X. arboricola* pathovars were recently elevated to the species rank as *X. guizotia*e and *X. populina* (Zarei et al., 2022).

The diagnostic procedures for *Xac* as recommended by EPPO rely on the observation of disease symptoms, microscopic examination of the symptomatic tissues, isolation of the pathogen from the plant material on common microbiological media for xanthomonads (Schaad et al., 2001), observation of colony morphology, biochemical, phenotypic, and pathogenicity assays (Lelliott and Stead, 1987; OEPP/EPPO, 2004). *Xac* also can be identified with serological methods following the procedures described in EPPO protocols (OEPP/EPPO, 2010b).

Molecular tools for rapid diagnosis of *Xac* colonies currently include methods specific for the genus *Xanthomonas* (Maes, 1993) and for the species *X. arboricola* (Pothier et al., 2011a). To identify *X. arboricola* isolates to the pathovar level, rep-PCR and partial sequence alignments are generally used (Tuang et al., 1999; Schaad et al., 2001; Scortichini et al., 2002; Parkinson et al., 2007; Young et al., 2008; Calić et al., 2009; OEPP/EPPO, 2010a; Puławska et al., 2010; Webber et al., 2020). Moreover, it was reported that primers designed for identification of *X. arboricola* pv. pruni (XapY17-F/XapY17-R) can also generate amplicons of some *Xac* strains (Pothier et al., 2011a; Webber et al., 2020).

Currently, there are no rapid and sensitive diagnostic tools for *Xac* (Prokić et al., 2012; Kałużna et al., 2021). The conventional methods are too labor-intensive and slow for routine detection and diagnosis, as complete diagnostic protocols can take several days. Additionally, the symptoms of bacterial blight of hazelnuts may be confused with anthracnose, a fungal disease caused by *Piggotia coryli* (Roberge ex Desm.) B. Sutton (Syn. *Gloeosporium coryli* (Roberge ex Desm.) Sacc.). Disease misidentification can lead to applying ineffective management methods and use of unwarranted chemical applications.

Recently, next-generation sequencing (NGS) and comparative genomics have developed as effective methods to provide information on pathogen population structures, create species specific markers, and characterize virulence or antibiotic resistance genes. The genomes and/or plasmids of several pathovars of *X*. *arboricola*, including *Xac*, have been sequenced (Pothier et al., 2011b; Pothier et al., 2011c; Ibarra Caballero et al., 2013; Garita-Cambronero et al., 2014; Cesbron et al., 2015; Higuera et al., 2015; Ignatov et al., 2015; Garita-Cambronero et al., 2016a; Garita-Cambronero et al., 2016b; Garita-Cambronero et al., 2016c; Harrison et al., 2016; López-Soriano et al., 2016; Garita-Cambronero et al., 2017; Retamales et al., 2017; Fernandes et al., 2018; Fu et al., 2018; Gétaz et al., 2018; Nuñez Cerda et al., 2021; Teixeira et al., 2021; Cuesta-Morrondo et al., 2022; D’Amico-Willman et al., 2022; Herbert et al., 2022; Kałużna and Pothier, 2022; Pothier et al., 2022). The available sequence data and the needs of the grower community and diagnostic laboratories prompted us to develop rapid, accurate and sensitive tools for the bacterial blight of hazelnut causal agent. We developed molecular tools for identification of *Xac* that could be used with several platforms, including conventional PCR, qPCR, and Loop-mediated isothermal AMPlification (LAMP), to facilitate adoption based on available laboratory equipment. We validated each of the tools using genomic DNA isolated from pure cultures of *Xac* and DNA isolated from artificially inoculated and field-infected plant material. These fast and accurate identification and detection methods will aid in the diagnosis and management of bacterial blight of hazelnut in nursery stock tissues, nurseries, and in both young and established orchards.

## 2 Materials and methods

### 2.1 Bacterial strains

*Xac* isolates and strains collected from different geographical regions (*n* = 60) were tested to validate all diagnostic assays. Additionally, a collection of type and non-type strains of all pathovars of *X*. *arboricola* species, other closely related *Xanthomonas* species (*n* = 30), and microorganisms (bacteria and fungi) isolated from symptomatic hazelnut and walnut tissues, i.e. *Pseudomonas* spp., *Pseudomonas avellanae*, *Sphingomonas* spp. and *Xanthomonas campestris* (*n* = 46) were included in assays (**Table 1**, **Supplementary Table S1**).

**Table 1:** Summary of *in vitro* primers specificity with the different *Xanthomonas arboricola* pv. corylina detection tools developed in this study.

Organism or material type	No. …	Conventional PCR						qPCR^1^						LAMP		
		Xac2.4‐1	Xac2.4‐4	XacPPU‐1	Xac45‐1	Xac45	XacPPU54630	Xac2.4‐2RT	Xac45‐1RT	Xac45‐2RT	Xac2.4‐3RT	Xac‐PPU54630	Xac‐reg 45	XacPPU‐1	New Xac2.4‐1	New Xac2.4‐2
*X. arboricola* pv. corylina (*n* = 60)	tested	42	42	42	42	36	36	28	28	28	28	23	23	23	23	23
	*positive*	*42*	*42^2^*	*42^3^*	*42*	*36*	*36*	*28*	*28*	*28*	*28*	*23*	*23*	*23*	*23*	*23*
Other *X. arboricola* pathovars (*n* = 27)	tested	22	22	22	22	6	6	22	23	22	22	17	17	22	22	22
	*positive*	*0*	*0*	*0*	*0*	*0*	*0*	*0*	*0*	*0*	*0*	*0*	*0*	*0*	*0*	*0*
Former *X*. *arboricola* pathovars (*n* = 3)	tested	2	2	2	2	1	1	2	2	2	2	2	2	2	2	2
	*positive*	0	0	0	0	0	0	0	0	0	0	0	0	0	0	0
*Pseudomonas avellanae* (*n* = 2)	tested	2	2	2	2	-^4^	–	2	2	2	2	2	2	2	2	2
	*positive*	*0*	*0*	*0*	*0*			*0*	*0*	*0*	*0*	*0*	*0*	*0*	*0*	*0*
HR^5^ negative *Pseudomonas* isolates from hazelnut (*n* = 9)	tested	8	8	8	8	–	–	8	8	8	8	2	2	8	8	8
	*positive*	*0*	*0*	*0*	*0*			*0*	*0*	*0*	*0*	*0*	*0*	*0*	*0*	*0*
*Sphingomonas* sp. non-pathogenic on hazelnut (*n* = 1)	tested	1	1	1	1	1	1	–	–	–	–	–	–	–	–	–
	*positive*	*0*	*0*	*0*	*0*	*0*	*0*									
*Xanthomonas campestris* non-pathogenic on hazelnut (*n* = 1)	tested	1	1	1	1	1	1	–	–	–	–	–	–	–	–	–
	*positive*	*0*	*0*	*0*	*0*	*0*	*0*									
HR positive *Pseudomonas* from walnut (*n* = 3)	tested	3	3	3	3	–	–	2	2	2	2	–	–	1	1	1
	*positive*	*0*	*0*	*0*	*0*			*0*	*0*	*0*	*0*			*0*	*0*	*0*
HR negative *Pseudomonas* and other hazelnut isolates (*n* = 8)	tested	7	7	7	7	–	–	8	8	8	8	–	–	8	8	8
	*positive*	*0*	*0*	*0*	*0*			*0*	*0*	*0*	*0*			*0*	*0*	*0*
HR negative *Pseudomonas* and other walnut isolates (*n* = 8)	tested	8	8	8	8	–	–	8	8	8	8	–	–	8	8	8
	*positive*	*0*	*0*	*0*	*0*			*0*	*0*	*0*	*0*			*0*	*0*	*0*
DNA from healthy plants (*n* = 5)	tested	5	5	5	5	–	–	5	5	5	5	–	–	5	5	5
	*positive*	*0*	*0*	*0*	*0*			*0*	*0*	*0*	*0*			*0*	*0*	*0*
Fungi isolated from diseased hazelnut (*n* = 4)	tested	4	4	4	4	–	–	4	4	4	4	–	–	–	–	–
	*positive*	*0*	*0*	*0*	*0*			*0*	*0*	*0*	*0*					
Fungi isolated from diseased walnut (*n* = 5)	tested	5	5	5	5	–	–	5	5	5	5	–	–	–	–	–
	*positive*	*0*	*0*	*0*	*0*			*0*	*0*	*0*	*0*					

^1^The first five columns for qPCR correspond to assays performed with SYBR^®^ Green I whereas the last column corresponds to a TaqMan™ assay.

^2^A smaller amplicon of 900 bp was observed with one strain instead of the 1,455 bp expected amplicon.

^3^Larger amplicons of 1,150 bp and 1,450 bp were observed with two strains instead of the 385 bp expected amplicon.

^4^“-” denotes not tested.

^5^HR: hypersensitivity reaction on tobacco leaves cv. ‘Samsun’.

Xanthomonads were grown on yeast extract nutrient agar (YNA) or yeast extract dextrose calcium carbonate (YDC; Schaad et al., 2001) and pseudomonads were cultured on King’s B medium (King et al., 1954) at 28°C for 24 to 48 h. The nine fungal isolates were grown on PDA (potato dextrose agar; Becton Dickinson, Sparks, MD, USA) at 24°C with an 8 h light and 16 h dark photoperiod.

### 2.2 DNA isolation from bacterial and fungal cultures

Genomic bacterial DNA was isolated using the Genomic Mini bacterial DNA Purification Kit (A&A Biotechnology, Poland), the DNeasy Mericon Food Kit (Qiagen, Hilden, Germany) or Whole Blood and Tissue kit (Qiagen, Germantown, MD, USA), according to the manufacturer’s instructions. The total fungal DNA was extracted from 100 mg of mycelia scraped from 10-day-old PDA cultures with the GeneMatrix Plant & Fungi DNA Purification Kit (EURx, Gdańsk, Poland) according to the manufacturer’s instructions. The quality and total DNA concentration was estimated with a NanoDrop ND-100 or NanoDrop 2000c (ThermoFisherScientific, Waltham, MA, USA).

### 2.3 Genome-informed target identification

DNA sequences from three *Xac* whole genome shotgun sequencing projects (WGS) (CFBP 1159^PT^, CFBP 2565 and NCCB 100457; GenBank WGS prefixes MDEA01, MDSJ01 and APMC02, respectively) was used for comparative genomic analysis. A ‘dual-BLASTn’ comparative genomics pipeline was applied to select 300-bp regions shared among these three target WGS (Schneeberger et al., 2017). After segmentation into 300 bp length fragments, duplicates were removed and *Xac* unique sequences were selected using BLASTn+ v.2.8.1 (Altschul et al., 1990; Camacho et al., 2009) analysis against the database derived from the three genomes. Regions obtained from this workflow were further checked for *Xac* specificity using online BLASTn searches against the *nr*/*nt* and *X*. *arboricola* and *Xanthomonas* WGS NCBI databases (accessed in July 2019). Finally, *Xac*-specific DNA markers were also confirmed in three recently released *Xac* complete genomes (CFBP 1159^PT^, CFBP 6600 and Xac 301; GenBank assemblies GCA_905220785.1, GCA_905220805.1, and GCA_905220715.1, respectively; Pothier et al., 2022).

### 2.4 Primer design and synthesis

**Figure 1:**
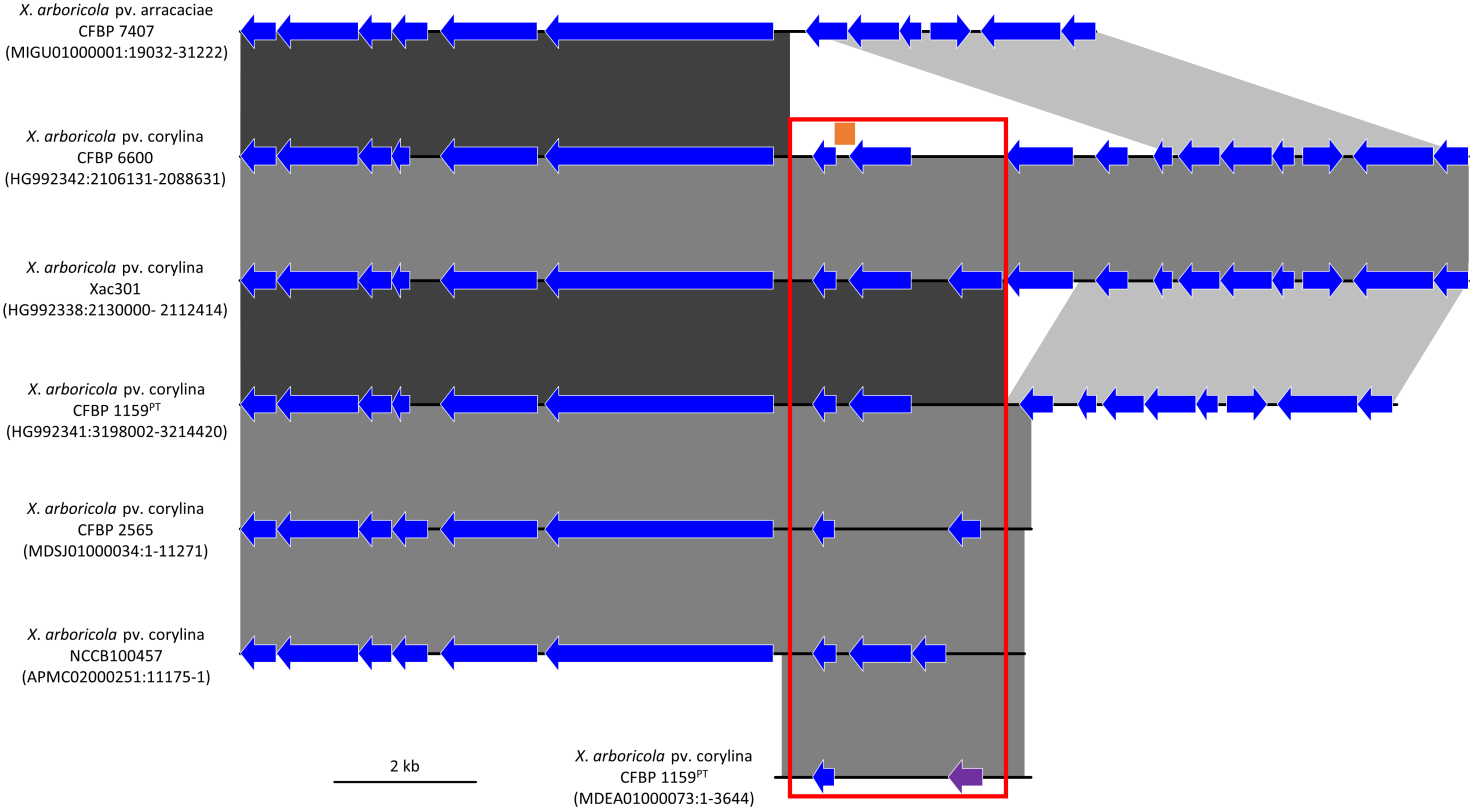
Comparison of the genetic environment of the *Xanthomonas arboricola* pv. corylina (*Xac*) specific DNA targets in six *Xac* draft and complete genomes and one draft *X*. *arboricola* pv. arracaciae draft genome. The 300 bp *Xac*-specific region called “target 45” is represented by an orange bar, the XaxcyCFBP1159_22010 singleton encoding the hypothetical protein “PPU54630” is displayed by a purple arrow, and the 2.4 kb region called “region 2.4” identified by comparative genomics is indicated by the red frame. Other CoDing Sequence (CDS) are shown with blue arrows, which do not denote any shared identity among the genomes. Regions with high DNA sequence identity between the genomes are represented with blocks using a black to grey scale with black representing the highest identity. The strain name is followed by the GenBank accession number and the location of the genomic region displayed.

Three *Xac*-specific regions and their associated primers were given ‘in-house’ names during analyses, the genome context of the regions is illustrated in **Figure 1**. These regions were used to design primers for: 1) conventional PCR, 2) qPCR (SYBR^®^ Green and TaqMan™), and 3) LAMP. The primers for conventional PCR and qPCR were designed using the PrimerSelect program of the LASERGENE package v.9 (DNASTAR, Madison, WI, USA) and Primer3Web v.4.1.0. (Untergasser et al., 2012). LAMP primers were designed using the online platform PrimerExplorer v.5 (Eiken Chemical Co., Ltd, Tokyo, Japan, http://primerexplorer.jp/lampv5e/index.html) also including loop primers (i.e. in total six primers) to speed up the LAMP reaction (Nagamine et al., 2002). Based on the regions selected (**Figure 1**), ten candidate primer sets were designed for conventional PCR (5, 2 and 3 primer sets based on the “region 2.4”, “PPU54630”, and “target 45” *Xac*-specific DNA markers, respectively), six for SYBR^®^ Green I qPCR (3, 1 and 2 primer sets based on the “region 2.4”, “PPU54630”, and “target 45” *Xac*-specific DNA markers, respectively), two for TaqMan™ qPCR (one primer pair based on the “region 2.4” and one based on the “target 45” *Xac*-specific DNA markers), and three for LAMP (two primer pairs based on the “region 2.4” and one based on the “PPU54630” *Xac*-specific DNA markers). The primers for the TaqMan™ qPCR were purchased HPLC purified since this effectively increases the melting temperature (*T_m_*) for shorter sequences, allowing for an overall shorter amplicon while remaining within temperature requirements. The TaqMan™ probes were designed with a 5′ FAM reporter dye and a 3’ BHQ-1 non-fluorescent quencher. Initially, the specificity of the primers, the TaqMan™ probe, and predicted amplicons to *Xac* were tested *in silico* with BLASTn searches against the *nr/nt* and WGS NCBI databases (accessed in July 2019). All these primer sets were then tested *in vitro* for specificity, sensitivity, and reproducibility during screening. Depending on the research institutions, primers were synthesized at Genomed S.A. (Warszawa, Poland), Invitrogen (ThermoFisherScientific, Waltham, MA, USA) and MilliporeSigma (Burlington, MA., USA).

### 2.5 Primer selection based on *in vitro* specificity analysis

The *in vitro* specificity of all primers was tested with purified genomic DNA of the bacteria and fungi listed in **Table 1** (detailed in **Supplementary Table S1**).

To exclude potential non-specific amplification of plant genomic DNA with the primers, total plant DNA was isolated from clean asymptomatic leaves of five hazelnut cultivars (cv. ‘Cosford’, cv. ‘Merveille de Bollwiller’, cv. ‘Garibaldi’, cv. ‘Webb’s Prize Cob’ and cv. ‘Hall’s Giant’) grown in a greenhouse. Total plant DNA was isolated from leaves using the GeneMATRIX Plant & Fungi DNA Purification Kit (EURx, Gdańsk, Poland), as well as the Genomic Mini DNA Extraction Kit (A&A Biotechnology, Gdynia, Poland) to isolate bacterial DNA. Both kits were used according to the manufacturer’s instructions with the following specifications concerning the starting material. To isolate plant DNA: 100 mg from hazelnut leaves were homogenized in liquid nitrogen in a cooled mortar and pestle and transferred to a 2 ml tube before addition of 400 μl lysis buffer L. For bacterial DNA isolation: 100 mg of crushed or cut leaf tissue was placed in 20 ml of PBS buffer, incubated for 1 h at 26°C with shaking (150 rpm), pelleted by centrifugation (5 min at 12,000 × *g*), and then re-suspended in 100 μl Tris EDTA (TE) buffer.

Three labs participated in the specificity validation of the assays: two assay development laboratories (Poland and Serbia) and one assay testing laboratory (USA).

The reactions were conducted according to the protocols established based on the optimization of all reagents and temperature gradient analysis performed separately for each primer pair. The amplification conditions for all the primers pairs/sets are listed in **Table 2**.

**Table 2:** Nucleotide sequences of specific primers developed in this study for the detection of *Xanthomonas arboricola* pv. corylina.

Assay target or code name^1^	Primer info^2^	Primer sequence 5’-3’	Amplicon length^3^ (bp)	MCA *T_m_* ^4^ (°C)
Conventional PCR				
Xac2.4-1	F	CCGCCACCATTTAGTACACGAGGAG	794	NA
	R	GGAGCCCGCGGAGATAGTTGC		
Xac2.4-4	F	TAATTCCAACTCCCCAAGCGTATC	1,455^5^	NA
	R	AATGAATTGGAGTGGTTGTTTAGG		
XacPPU-1	F	TCCCAACACTAAGTCTTCAACATC	385^6^	NA
	R	GGTGCAGGTGGGAGGTGGTAAC		
Xac45-1	F	TTCCTCAATGCGGGCCAGTAATGTC	197	NA
	R	ATAGTGATAATGAGGTGGCAGTCG		
Xac45	F	CCAGTCTCACCCAACGTCAGA	198	NA
	R	TGTCGTGGAATCAACCTGATGTG		
XacPPU54630	F	CACCAGAAAAGCAGGGCCATAAC	159	NA
	R	GGCAATGGAAGGACGTCTAGG		
qPCR SYBR^®^ Green I				
Xac2.4-2RT	F	AGCAGGGCCATAACTTCTTG	170	81.5
	R	ATATACACCCCTTTTTGGATGG		
Xac45-1RT	F	CTTGCCCAGCCCCCAGTC	104	84.5
	R	TATGAACAACGTACCGCAGATG		
Xac45-2RT	F	AAGTGCTTGCAAATAATAAATC	88	81.5
	R	TGTCGTGGAATCAACCTG		
Xac2.4-3RT	F	GCCACCATTTAGTACACGAGGAGTTC	102	81.0
	R	TATTTCGGTAGAGCTAGTCGGTTGTC		
qPCR TaqMan™				
Xac-reg45	F	CCAGTCTCACCCAACGTCAGA	198	NA
	R	TGTCGTGGAATCAACCTGATGTG		
	P	FAM-CATGATCATTCCTCAATGCG-BHQ-1		
Xac-PPU54630	F	CACCAGAAAAGCAGGGCCATAAC	159	NA
	R	GGCAATGGAAGGACGTCTAGG		
	P	FAM-TAATTAACCAAGCCATCGCC-BHQ-1		
LAMP				
XacPPU-1	F3	CGAAAAAAATAAGGAAACTTCACC	(214)^7^	84
	B3	ATTCATAGCGCCACGATA		
	FIP	GGATGGCAATGGAAGGACGTCACCCCCTATCTCCCTC		
	BIP	TAGAAAAGAAAGAAAGCTATCCGCTAAATGAATTGGAGTGGTTGTT		
	LF	AGGTTAGCCCTTCAGGTACTC		
	LB	ACTAGGCTCATCTATTACCCTAGTT		
Xac2.4-1	F3	CGAAAAAAATAAGGAAACTTCACC	(214)	83.5
	B3	ATTCATAGCGCCACGATA		
	FIP	TACACCCCTTTTTGGATGGCAATCCCTATCTCCCTCATGAGTAC		
	BIP	TAGAAAAGAAAGAAAGCTATCCGCTAAATGAATTGGAGTGGTTGTT		
	LF	GAAGGACGTCTAGGTTAGCCCTTCA		
	LB	ACTAGGCTCATCTATTACCCTAGTT		
Xac2.4-2	F3	ATTCCTGAGGACTAGGCACT	(186)	87.5
	B3	CTTTGAGACGCGCTGTCG		
	FIP	TTGTGGTGAAGAACCGCCGTATCTGATCATCGAGGGACCCG		
	BIP	GCAAGGAAACTCTGGCAACGGATGCGCTAGGCATATTTGGTG		
	LF	GGAGGTGGTCTTTATAATGCTGG		
	LB	AAAGTTTCAGCCGAGGCAAA		

^1^Primer names begin with Xac45, Xac2.4, or XacPPU to indicate targeted genomic regions (target 45, region 2.4, or PPU54630, respectively) shown in **Figure 1**. RT at the end of the primer code name stands for real-time.

^2^Primer information is abbreviated as follows, F, forward primer; R, reverse primer; P, probe; F3, forward outer primer; B3, backward outer primer; FIP, forward inner primer; BIP, backward inner primer; LF, forward loop primer; LB, backward loop primer.

^3^Expected amplicon length based on the complete genome of *X. arboricola* pv. corylina CFBP 1159^PT^ (GenBank accession number HG992341) and amplicon size commonly observed during *in vitro* tests. Depending on the assay, a few strains produced an amplicon with a different size as indicated below in footnotes 5 and 6, and with more details in **Supplementary Table S1**.

^4^Specific melting temperature observed during melting curve analysis. NA, not applicable.

^5^A smaller amplicon of 900 bp was observed with one strain.

^6^Larger amplicons of 1,150 bp and 1,450 bp were observed with two strains.

^7^Parentheses indicate the predicted size (bp) of the region targeted by the F3 and B3 primers in LAMP assays.

Amplification reactions with the four selected primer pairs for conventional PCR were conducted in a Biometra T3000 thermocycler (Biometra, Göttingen, Germany) in Poland, in a Thermo Cycler 2720 (Applied Biosystems, USA) in Serbia, and a Veriti 96-well Thermal Cycler 9902 (Applied Biosystems, USA) in the USA. The total amplification reaction mixtures for primers in 15 μl of volume included: 10 to 15 ng of DNA, 0.4 U of DreamTaq DNA Polymerase (ThermoFisherScientific, Waltham, MA, USA), 1× reaction DreamTaq Green buffer, 0.15 mM each dNTPs and 0.7 mM of each primer. The amplicons obtained in individual reactions for each primer pair were separated in 1.5% agarose gels in 0.5× TBE buffer (0.045 M Tris-boric acid, 0.001 M EDTA, pH 8.0) (Sambrook et al., 1989). To confirm the size of the obtained product O’GeneRuler100-bp DNA Ladder Plus (ThermoFisherScientific, Waltham, MA, USA) was used. Gels were stained in an ethidium bromide solution (0.5 μg ml^-1^) and obtained products were visualized under UV irradiation.

SYBR^®^ Green I qPCRs were conducted in a Bio-Rad CFX96 (Bio-Rad, Hercules, CA, USA) with SsoAdvanced™ Universal SYBR^®^ Green Supermix (Bio-Rad, Hercules, CA, USA) in Poland or a Mic qPCR Cycler (Bio Molecular Systems, Australia) in Serbia. The reaction mixture in 20 μl of total volume included 1× reaction SYBR^®^ Green Supermix and 0.5 mM of each primer from the following primer sets: Xac2.4-2RT, Xac45-1RT, Xac45-2RT, Xac2.43RT, and 10 ng of DNA. The PCR programs for all above-listed primers are given in **Table 3**. The specificity of amplification products was verified by a melting curve analysis using a progressive denaturation of products at a rising temperature (**Table 3**). Specific melting temperatures observed are indicated in **Table 2**.

**Table 3:** Amplification conditions for the primers pairs/sets designed and used in this study.

Detection tool	Assay target	Reaction conditions
**Conventional PCR**	Xac2.4-1	95°C for 4 min, 30× (94°C for 35 s, 63°C for 45 s, 72°C for 1 min), 72°C for 10 min
	Xac2.4-4	95°C for 4 min, 35× (94°C for 35 s, 58°C for 45 s, 72°C for 1 min), 72°C for 10 min
	XacPPU-1	95°C for 4 min, 30× (94°C for 30 s, 61°C for 40 s, 72°C for 55 s), 72°C for 10 min
	Xac45-1	95°C for 4 min, 30× (94°C for 25 s, 61°C for 35 s, 72°C for 50 s), 72°C for 7 min
	Xac45	95°C for 2 min, 30× (94°C for 30 s, 53°C for 30 s, 68°C for 45 s), 68°C for 5 min
	XacPPU54630	95°C for 2 min, 30× (95°C for 30 s, 53°C for 30 s, 68°C for 45 s), 68°C for 5 min
**qPCR SYBR^®^ Green I**	all primer pairs	98°C for 2 min, 35× (95°C for 10 s, 60°C for 20 s), 65→95°C with +0.01°C s^-1^
**qPCR TaqMan™**	all primer sets	95°C for 10 min, 40× (95°C for 10 s, 55°C for 40 s)
**LAMP**	all primer sets	50× (63°C for 30 s), 65→95°C with +0.01°C s^-1^

The validation of the TaqMan™ qPCR was also done in Poland. The sequence of probes and primers are indicated in **Table 2**. Reactions were conducted in a Bio-Rad CFX96 (Bio-Rad, Hercules, CA, USA) using the amplification conditions in **Table 3**. The TaqMan™ qPCR assays were carried out in a 10 μl total reaction mixture containing 1 μl of template DNA, 0.25 μl of primers Xac-PPU54630-F and Xac-PPU54630-R (0.25 μM final concentration of each), 0.15 μl of probe Xac-PPU54630-P (0.15 μM final concentration), 1× TaqMan™ Fast Universal PCR Master Mix (Applied Biosystems, USA).

Loop‐mediated isothermal amplifications were performed on a Bio-Rad CFX96 (Bio-Rad, Hercules, CA, USA) in Poland. The reactions mixture carried out in a total volume of 20 μl contained 1× Isothermal Mastermix (OptiGene, Horsham, UK) and primers at the final concentrations as follows: outer primers F3/B3 0.2 μM each, inner primers FIB/BIP 0.8 μM each and loop primers 0.4 μM each. Fluorescence was detected on the FAM channel. The LAMP reaction mixtures were run according to conditions detailed in **Table 3**.

### 2.6 Limits of detection of DNA- and crude bacterial cell-based assays

The limits of detection (LoD) of all the DNA-template based assays were tested with 10-fold dilutions series prepared in TE buffer using bacterial genomic DNA isolated from pure cultures of CFBP 1159^PT^ and Xac 301. The dilution series ranged from ~10 ng μl^-1^ to 0.1 fg μl^-1^ based on the initial concentrations determined with a NanoDrop ND-100 (ThermoFisherScientific, Waltham, MA, USA). Additionally, bacterial genomic DNA was independently extracted from pure bacterial cultures of these two same strains using known bacterial concentrations ranging from ~10^8^ to 10^0^ CFU ml^-1^ as described in Kałużna et al. (2016).

For the crude bacterial cell-template based assays, 100 μl of different concentrations of aqueous suspensions of strain Xac 301 were added to 100 mg of crushed/cut fragments of leaves or stems. Then DNA was isolated from these ‘heterogeneous suspensions’ according to the methodology described by Kałużna et al. (2016).

For the PCR-based assays, the efficiency (*E*) was calculated from the slope (*S*) of the standard curve generated for each run using the following equation *E* = 10^(−1/^ *^S^* ^)^ with *E* = 2 corresponding to 100% efficiency (Ramakers et al., 2003).

### 2.7 Validation of assays on artificially and naturally infected hazelnuts

To test the usefulness of designed primers, positive controls for *in planta* detection were obtained from artificially inoculated hazelnut cvs. ‘Cosford’ and ‘Merveille de Bollwiller’ (two samples from each cultivar) maintained in a greenhouse, as well as from naturally infected material obtained from orchards (two samples). For artificial inoculation of the hazelnut cultivars, a 48-h culture of Xac 301 grown on YNA medium was suspended in sterile water (10^8^ and 10^7^ CFU ml^-1^) and infiltrated into hazelnut leaves with a needleless syringe and/or injected into green shoots using a hypodermic needle (0.7×30mm) attached to a syringe. Four to six weeks post-inoculation, symptomatic plant tissue was harvested. Leaf samples were rubbed for 10 s on both sides with a cotton-swab soaked in 70% ethanol. A sample consisting of three 1-cm^2^ segments including the lesion border was collected, crushed, and suspended in 1 to 2 ml sterile PBS for 15 min. We then tested two DNA extraction methods on the tissue macerate. In the first one, 10 μl of the plant macerate was added to 190 μl of TE buffer, boiled for 10 min at 100°C, and then centrifuged for 5 min at 9,500 × *g*. In the second approach, 10 μl of the plant macerate was added to 90 μl of TE buffer and total DNA was isolated using the Genomic Mini DNA Extraction Kit (A&A Biotechnology Gdynia, Poland) according to the manufacturer’s instructions. The boiled extract and purified DNA extract were used as templates in molecular assays. To confirm the infection by *Xac*, especially from naturally infected plant material, bacterial colony isolation was done simultaneously by plating on YNA medium.

## 3 Results

### 3.1 Genome-informed *Xac*-specific targets

The *in silico* analysis resulted in the detection of a highly conserved, *Xac*-specific sequence of 300 bp called “target 45” that had no hit with other bacteria in the database. A 2,440 bp genomic region called “region 2.4” encompassing “target 45” was identified after performing the comparative genomic analysis of target 45 in the six *Xac* whole genomes available (**Figure 1**). Region 2.4 located on the chromosome corresponds to an insertion in *Xac* that was not present in other *X*. *arboricola* pathovars, such as pv. arracaciae (**Figure 1**). The annotations for this region varied slightly between the different *Xac* genomes, but the region contains between two and three singletons that encode hypothetical proteins. A 494 bp singleton located within “region 2.4” in *Xac* CFBP 1159^PT^ (locus_tag XaxcyCFBP1159_22010) and annotated as encoding the hypothetical protein PPU54630 was used for the further development of *Xac*-specific assays.

### 3.2 Candidate primer sets and *Xac* assays development

Out of the candidate primer sets designed for all three detection techniques, a few sets were discarded from further analysis due to the presence of non-specific products that persisted, even after adjusting annealing temperatures. After initial laboratory testing, we focused on validation and testing of six primer pairs for conventional PCR, four primer pairs for qPCR with SYBR^®^ Green I, two primer pairs for TaqMan™ qPCR, and three primer pairs for LAMP. The sequences of these primer sets are reported in **Table 2**. A primer BLASTn analysis of selected primers showed no full similarity to any sequences of bacterial plant pathogens in GenBank in July 2019. This *in silico* result was also confirmed on 15 May 2023 with a final primer check performed in the course of writing this article.

### 3.3 Primers specificity for *Xac* in conventional, qPCR and LAMP *in vitro* and *in planta*

The genomic DNA of the 60 *Xac* strains was selectively amplified with all the primers developed for the different assays. No amplification was observed for the bacterial and fungal genomic DNA not belonging to the *Xac* pathovar (**Table 1**). Similarly, no amplification was observed with DNA templates obtained from clean, asymptomatic leaves of five hazelnut cultivars using two DNA extraction kits.

The PCR assays using primers designed for conventional PCR gave amplicons ranging from 197 bp to 1,455 bp depending on the primer pairs used (**Table 2**, **Supplementary Table S1**). The six primer sets designed for conventional PCR generated a single amplicon of the size predicted by genome analyses for nearly all the 60 strains of *Xac* evaluated (**Table 1**). Although, during validation of conventional PCR reactions on the JL26xx strains of *Xac* collected in Oregon, amplicons with an unexpected size were observed with *Xac* strain JL2600 with primers Xac2.4-4 and XacPPU-1. For *Xac* strain JL2600, the amplicon observed for primer pair for Xac2.4-4 was 1,166 bp instead of 1,455 bp and the amplicon for the primer pair for XacPPU-1 was 1,450 bp instead of 390 bp. The other conventional PCR primer pairs generated the predicted amplicon size for *Xac* strain JL2600. Conventional PCR reactions for all the other *Xac* strains in the JL26xx series (**Supplementary Table S1**) returned the expected amplicon size for each of the primer pairs.

In the SYBR^®^ Green I qPCR assays, DNA from the *Xac* strains resulted in a positive reaction. However, non-specific, false-positive results after 28 cycles for a few bacteria not belonging to the *Xanthomonas* genus were observed when using the primers Xac45-1. Nonetheless, these non-specific amplicons were excluded based on the results of melting curve analysis i.e., having different melting temperature than the target product. The amplicons ranged from 88 bp to 170 bp and melting curve analysis performed on these specific products revealed a single peak characteristic of their already introduced line 492 *T_m_* as reported in **Table 2**.

In the TaqMan™ qPCR, two primer sets designed resulted in a positive reaction for the tested DNA from the *Xac* strains tested (**Table 1**, **Supplementary Table S1**) and no product were observed in case of testing of bacterial and fungal genomic DNA not belonging to the *Xac* pathovar nor DNA templates obtained from clean, asymptomatic leaves of five hazelnut cultivars.

In the LAMP assays, DNA of the *Xac* strains gave a positive reaction as expected and no amplification was observed with DNA of other isolates. The *T_m_* of products amplified using the LAMP primers are provided in **Table 2**.

Specificity, sensitivity, and efficiency of the *Xanthomonas arboricola* pv. corylina specific assays based on the organisms evaluated in this study are reported in **Table 4**.

**Table 4:** Specificity, sensitivity, and efficiency of the *Xanthomonas arboricola* pv. corylina specific assays based on the organisms evaluated in this study.

	Conventional PCR						qPCR^1^						LAMP		
	Xac2.4‐1	Xac2.4‐4	XacPPU‐1	Xac45‐1	Xac45	XacPPU54630	Xac2.4‐2RT	Xac45‐1RT	Xac45‐2RT	Xac2.4‐3RT	Xac‐PPU54630	Xac‐reg 45	XacPPU‐1	New Xac2.4‐1	New Xac2.4‐2
*N* ^2^	110	110	110	110	45	45	94	95	94	94	46	46	79	79	79
*N_TP_*	42	42	42	42	36	36	28	28	28	28	23	23	23	23	23
*N_TN_*	68	68	68	68	9	9	66	67	66	66	23	23	56	56	56
*N_FP_*	0	0	0	0	0	0	0	0	0	0	0	0	0	0	0
*N_FN_*	0	0	0	0	0	0	0	0	0	0	0	0	0	0	0
Assay sensitivity (%)	100	100	100	100	100	100	100	100	100	100	100	100	100	100	100
Assay specificity (%)	100	100	100	100	100	100	100	100	100	100	100	100	100	100	100
Test efficiency (%)	100	100	100	100	100	100	100	100	100	100	100	100	100	100	100

^1^The first five columns for qPCR correspond to assays performed with SYBR^®^ Green I whereas the last qPCR column corresponds to a TaqMan™ assay.

^2^ *N*, total number of samples tested; *N*_TP_, true positive samples; *N*_TN_, true negative samples; *N*_FP_, false positives and *N*_FN_, false negatives.

The LAMP and both qPCR assays confirmed identity of verified *Xac* strains. Results for LAMP and qPCR platforms were obtained in less than 1 h.

### 3.4 Limits of detection of DNA- and crude bacterial cell-based assays

The sensitivity and detection limit of the *Xac* target DNA varies not only between the detection systems developed but also depending on the primer sets used. For four primer pairs designed for conventional PCR, 100 fg of genomic DNA generated a visible amplicon with primer pairs Xac2.4-4, and XacPPU-1; ~1 pg genomic DNA for primer pair Xac2.4-1; and 10 pg was detected with primer set Xac45-1. When crude, boiled bacterial cell templates were tested, the LoD was 1.8 × 10^1^ CFU per reaction for Xac2.4-1 and XacPPU-1 primer sets, 1.8 × 10^0^ CFU per reaction for Xac2.4-4 primer sets, 1.8 × 10^2^ CFU per reaction for XacPPU54630 primer sets, and 1.8 × 10^3^ CFU per reaction for Xac45-1 primer sets.

The LoD was lowered by 10^1^ when using the primer pairs Xac2.4-1, Xac2.4-4, and XacPPU-1 to detect *Xac* in plant tissue macerates that contained the pathogen; for the primer pairs XacPPU54630 and Xac45-1 the LoD remained the same with or without plant tissues.

**Figure 2:**
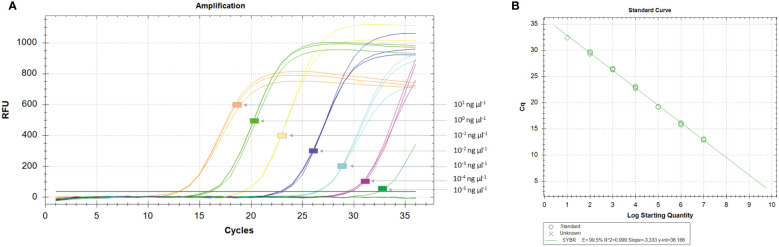
Determination of the limit of detection (LoD) of the *Xanthomonas arboricola* pv. corylina (*Xac*) qPCR SYBR^®^ Green I assay **(A)** and standard curve **(B)**. The representative amplifications were obtained with the Xac2.4-3RT SYBR^®^ Green I assay using 10-fold dilutions (three technical replicates) of genomic DNA of known concentrations isolated from pure cultures of strain Xac 301. The efficacy reaction *E*, coefficient of determination (*R* ^2^), slope and regression curve equations (*y*) were evaluated using the CFX Manager Software v.3.1 (Bio-Rad, Hercules, CA, USA).

Among qPCR primers designed for SYBR^®^ Green I, two primer sets (Xac2.4-3RT and Xac45-1RT) detected 1 fg of *Xac* genomic DNA (**Figure 2**), however the two other primer sets (Xac2.4-2RT and Xac45-2RT) detected about 10 fg of *Xac* genomic DNA. When crude boiled bacterial templates were tested, the limit of detection was 1 × 10^0^ CFU per reaction. The same decrease of sensitivity as noticed for conventional PCR (lowered by 10^1^) for boiled bacterial preparations and in combinations of plant tissues and bacteria. Parameters of the four qPCR SYBR^®^ Green I assays are reported in **Table 5**.

**Table 5:** Parameters of the four qPCR SYBR^®^ Green I assays evaluated through the analysis of standard curves generated with serial dilutions of genomic DNA extracts from *X. arboricola* pv. corylina CFBP 1159^PT^ and Xac 301 as templates.

qPCR code name	*E* (%)^1^	*R* ^2^2^^	*S* ^3^	*Y* = *int* ^4^
**Xac2.4-2RT**	101.9	0.996	-3.276	35.671
**Xac45-1RT**	102.6	0.991	-3.261	36.210
**Xac45-1RT**	99.7	0.998	-3.330	36.915
**Xac2.4-3RT**	99.5	0.999	-3.333	36.166

^1^ *E* stands for PCR efficiency.

^2^ *R^2^* is a measure of data linearity among technical replicates (*n* = 3) of serial dilutions.

^3^The slope (*S*) of the log–linear phase of the amplification reaction is a measure of reaction efficiency.

^4^ *Y* = *int* represents the cycle threshold (*C_t_*) value where the curve crosses the *y*-axis.

For TaqMan™ qPCR, the primer sets Xac-reg45 and Xac-PPU54630 detected 80 and 8 pg of *Xac* genomic DNA, respectively. When crude boiled bacterial templates were tested, the limit of detection was 2 × 10^1^ CFU per reaction for primer set Xac-PPU54630 and 2 × 10^3^ CFU per reaction for primer set Xac-reg45.

When determining the sensitivity of LAMP primers, we detected 1 pg of purified genomic DNA from *Xac* isolates. When boiled bacterial cell templates were tested, the LoD was 1 × 10^0^ CFU per reaction for the XacPPU-1 primer set and 1 × 10^1^ CFU per reaction for the Xac2.4-1 and Xac2.4-2 primer sets. In purified DNA isolated from plant material combined with bacteria, the LoD was 1 × 10^3^ CFU per reaction for all the primers tested.

### 3.5 Performance of the different detection tools on tissues from artificially inoculated and naturally infected hazelnuts

The detection of *Xac* in artificially inoculated plant material was done with four conventional primer sets (Xac2.4-1, Xac2.4-4, XacPPU-1 and Xac45-1), all SYBR^®^ Green I qPCR (*n* = 4) and all LAMP primer sets (*n* = 3). All the primer sets used in the different platforms returned positive results for detection of *Xac* when the DNA was isolated using the kit procedure. Nonetheless, when the volume of the template of purified genomic DNA (µl or concentration per reaction) significantly increased, detection was decreased. Correspondingly, a one-tenth dilution of the purified genomic DNA template added to plant tissues allowed for consistent detection of *Xac*.

With all four conventional PCR primer sets, *Xac* was not detected when the assays were performed on DNA templates obtained *via* the boiling procedure of plant macerate (plants artificially or naturally infected). Because this was not the case with templates consisting of purified genomic DNA, we suspect that the boiling procedure did not eliminate possible plant inhibitors. The assays also remained negative when a tenfold dilution of the extracts was tested. For the SYBR^®^ Green I qPCR primer sets, *Xac* was detected in DNA isolated with both procedures independent of the template DNA concentration. With the LAMP XacPPU-1, Xac2.4-1 and Xac2.4-2 primer sets, *Xac* was always detected with purified genomic DNA preparations. In case of DNA extracted by boiling, templates with only 0.5 and 1 µl of undiluted extract was detected.

## 4 Discussion

Based on a comparative genomics approach using five publicly available *Xac* genomes (Ibarra Caballero et al., 2013; Merda et al., 2017; Pothier et al., 2022) and several bacterial genomes from NCBI GenBank, we successfully identified unique DNA targets and designed highly specific tools capable of identifying *Xac* in pure culture and culture-independent *in planta* detection. We developed four different systems for conventional PCR and qPCR, as well as a LAMP protocol for the rapid and specific detection of *Xac*. This ensures a wide application of the developed detection methods, depending on the equipment or preferences of scientists, diagnosticians, inspectors, and producers. In addition, these methods offer an advantage over conventional testing as bacteria do not need to be cultured prior to detection (Palacio-Bielsa et al., 2009). This could prove especially useful in the context of screening nursery material for latent infections, which would otherwise go undetected and become a source of primary infection in the field. For regions where new hazelnut acreage is rapidly increasing, such as Serbia and Chile (Lamichhane et al., 2012; Obradović et al., 2010), disease-free planting material is a critical first step to keep *Xac* disease pressure low.

Historically, hazelnut bacterial blight diagnostics have relied upon a combination of classical microbiology, serology, and molecular techniques (Schaad et al., 2001; OEPP/EPPO, 2004; Pothier et al., 2011a; Prokić et al., 2012; Kałużna et al., 2021). While recommended by EPPO, these methods are time consuming and risk misdiagnosis (Prokić et al., 2012). Moreover, none of them provide a LoD. For example, the biochemical features of Polish strains differ from those described in the EPPO standard. As a result, the recommended phenotype testing methods are not applicable to strains from the Polish climatic zone (Puławska et al., 2010). Similar issues have emerged when conducting the recommended procedure of sequencing housekeeping genes to identify pathovars within *X. arboricola*. The multilocus sequence analysis within this species showed that using a restricted number of housekeeping gene loci did not have sufficient discriminatory power to differentiate isolates of *Xaj* and *Xac* into unique groupings. Moreover, the use of partial *gyrB* sequences alone cannot discriminate *Xaj* and *Xac* from *Xap* (Kałużna et al., 2014; Fischer-Le Saux et al., 2015; Webber et al., 2020). The molecular tools reported herein overcome these sub-species diagnostic shortcomings.

The success of our work is based on comparing the genomes of all *X*. *arboricola* pathovars and related *Xanthomonas* species (Zarei et al., 2022), which allowed for the selection of a highly specific regions for *Xac*. The specificity of the region identified within the six genomes used for *in silico* development (three WGS and three complete genomes from five *Xac* strains) also was confirmed when tested with BLASTn analysis against three additional complete *Xac* genomes released after our assay development (namely: A7, assembly ASM1814170v1; IVIA 3978, assembly ASM2337497v1; CFBP 1846, assembly ASM2337499v1; data not shown). The success of our approach likely benefitted from the large number of genomes available for the *X*. *arboricola* species (about 100 genomes at the time of *in silico* development) thus allowing the development of assays at a sub-species level. The designed diagnostic tools allowed the detection of *Xac* genotypes from different worldwide geographical origins. A total of 60 *Xac* strains originating from eight countries in two continents and collected over 20 different years spanning the period 1939-2020 was tested successfully. The only exception was a result for the conventional PCR primer set Xac2.4-4 and XacPPU-1 when screening a set of *Xac* isolates from the United States. *Xac* isolate JL2600 amplified successfully, which indicates a *Xac* positive result, but the resulting amplicon was larger than expected. This result is particularly surprising because the dendrogram constructed using the concatenated partial sequences of *rpoD* and *gyrB* (Webber et al., 2020), had strain JL2600 clustered together with strain JL2606, an isolate for which the expected amplicon size was obtained. All other *Xac* strains (e.g., JL2610) belonging to the other *Xac* cluster described in the work by Webber et al. (2020) gave the expected amplicon size. This result reaffirms that validation testing of a comprehensive collection of strains, preferentially in different laboratories, is very important when developing novel identification and detection systems. Importantly, *Xac* specificity was confirmed by all detection assays and none of the non-*X*. *arboricola* pathovars tested returned a positive amplicon, which has happened in previous studies (Palacio-Bielsa et al., 2011; Pothier et al., 2011a; Fernandes et al., 2017). Also, none of the genomic DNA of *Pseudomonas*, other plant pathogenic and nonpathogenic bacteria, or fungi isolated from hazelnut and walnut gave a positive signal in the assays. In addition, no amplification was observed from DNA isolated from asymptomatic plants of different *C. avellana* cultivars, which means that the designed primers did not react with the hazelnut genome or its microbiota.

The methods and tools developed here can be applied for specific, reliable detection of *Xac* in infected plant material. Not having to first isolate and purify the pathogen significantly shortens the time required for diagnosis. All methods presented in this study allow for direct amplification of *Xac* DNA present in plant material. However, we observed that direct detection of DNA templates extracted by boiling can give false negative results, most likely due to the presence of inhibitory compounds. This phenomenon has already been observed with culture-independent detection of other pathogens *in planta* (De Boer et al., 1995; López et al., 2009; Palacio-Bielsa et al., 2009; Gétaz et al., 2017) and did not occur with the qPCR assays. The use of a DNA extraction kit eliminated putative DNA polymerase inhibitors and supports the finding of López et al. (2009) that the purification methods used should be evaluated for each combination of tested pathogen and plant before establishing and recommending the procedure for routine detection. Therefore, a DNA extraction kit is recommended for detection of *Xac* DNA in hazelnut tissues.

The LoD of the different assays was satisfactory for all the primer sets and allowed detection of between 1 pg to 10 fg per reaction or 1 × 10^0^ to 1 × 10^3^ CFU per reaction, with the highest sensitivity obtained for qPCRs. The qPCR procedure turned out to be the fastest of the protocols developed, with the whole reaction and melting curve analysis taking about 1 hour. The high sensitivity of these assays is especially important in the case of naturally infected plant material with low populations of the pathogen. LoD values, similar to the ones obtained in this study were observed previously during the development of detection methods for other *X*. *arboricola* pathovars (Palacio-Bielsa et al., 2011; Pothier et al., 2011a; Fernandes et al., 2017), as well as for diagnostics of other plant pathogenic bacteria from other species or genus, e.g., *Pseudomonas morsprunorum* race 1 and 2 (Kałużna et al., 2016), *P. syringae* pv. actinidiae (Gallelli et al., 2014), *X. campestris* pv. campestris (Eichmeier et al., 2019).

The *Xac* detection systems developed allow for quick and reliable determination of host plant infection without the requirement for isolation of the bacterial pathogen. These assays also can be used to improve our knowledge of this pathogen, such as exploration of other host plants and natural reservoir(s). Even in the presence of potential plant inhibitors, the sensitivity of the assays remained high and sample-to-result times ranged from 5 to 6 hours for conventional PCR down to 1 to 2 hours for qPCR and LAMP assays. So far, this group of molecular assays is the first such methods available for rapid detection of the *Xac* pathogen directly from plant material.

## Data availability statement

Publicly available datasets were analyzed in this study. This data can be found here: https://www.ncbi.nlm.nih.gov/datasets/genome/GCF_002939845.1, https://www.ncbi.nlm.nih.gov/datasets/genome/GCA_002940125.1, https://www.ncbi.nlm.nih.gov/datasets/genome/GCA_000355635.2, https://www.ncbi.nlm.nih.gov/bioproject/PRJEB42844.

## Author contributions

MK: conceptualization, funding-acquisition, investigation, methodology, visualization, writing-original-draft, writing-review-editing. AP: investigation, software, writing-review-editing. AO: funding-acquisition, writing-review-editing. WW: investigation, writing-review-editing. VS: investigation, writing-review-editing, funding-acquisition. JP: conceptualization, funding-acquisition, software, visualization, writing-original-draft, writing-review-editing.
