# Supplementary material for: Specific and sensitive detection tools for Xanthomonas arboricola pv. corylina, the causal agent of bacterial blight of hazelnut, developed with comparative genomics
Source: Front Plant Sci. 2023 Sep 13;14:1254107. doi: 10.3389/fpls.2023.1254107 (PMC10535005; doi:10.3389/fpls.2023.1254107)
Supplement: Supplementary file 1 [file DataSheet_1.pdf]

Supplementary Material

belonging to

Specific and sensitive detection tools for *Xanthomonas arboricola* pv. corylina, the causal agent of bacterial blight of hazelnut, developed with comparative genomics

Monika Kałużna[1*], Andjelka Prokić[2], Aleksa Obradović[2], William A. Weldon[3], Virginia O. Stockwell[4], Joël F. Pothier[5*]

1 Supplementary Tables

Supplementary Table 1. The origin of the studied *Xanthomonas arboricola* strains, other bacteria from hazelnut and walnut as well as other bacteria and fungi tested during *in vitro* primers specificity with the different *X. arboricola* pv. corylina detection tools developed in this study.

Supplementary Table 1. The origin of the studied *Xanthomonas arboricola* strains,other bacteria from hazelnut and walnut as well as other bacteria and fungi tested during *in vitro* primers specificity with the different *X. arboricola* pv. corylina detection tools developed in this study.

Organism name or type	Strain or isolate[1]	Geographic origin	Year	Conventional PCR						qPCR[2]						LAMP		
				Xac2.4-1	Xac2.4-4	XacPPU-1	Xac45-1	Xac45	XacPPU54630	Xac2.4-2RT	Xac45-1RT	Xac45-2RT	Xac2.4-3RT	Xac-PPU54630	Xac-reg 45	XacPPU-1	New Xac2.4-1	New Xac2.4-2
X. arboricola pv. corylina	CFBP 1159[PT]	Oregon, USA	1939	+	+	+	+	nt	nt	+	+	+	+	+	+	+	+	+
	LMG 688		1964	+	+	+	+	nt	nt	+	+	+	+	+	+	+	+	+
	290	Pamietna, łódzkie, PL	2007	+	+	+	+	nt	nt	+	+	+	+	+	+	+	+	+
	295			+	+	+	+	nt	nt	+	+	+	+	+	+	+	+	+
	296			+	+	+	+	nt	nt	+	+	+	+	+	+	+	+	+
	297			+	+	+	+	nt	nt	+	+	+	+	+	+	+	+	+
	299			+	+	+	+	nt	nt	+	+	+	+	+	+	+	+	+
	300			+	+	+	+	nt	nt	+	+	+	+	+	+	+	+	+
	301			+	+	+	+	nt	nt	+	+	+	+	+	+	+	+	+
	302b			+	+	+	+	nt	nt	+	+	+	+	+	+	+	+	+
	303			+	+	+	+	nt	nt	+	+	+	+	+	+	+	+	+
	305			+	+	+	+	nt	nt	+	+	+	+	+	+	+	+	+
	2034-1	Lipnik, świętokrzyskie, PL	2020	+	+	+	+	nt	nt	+	+	+	+	+	+	+	+	+
	2034-2			+	+	+	+	nt	nt	+	+	+	+	+	+	+	+	+
	2035-1			+	+	+	+	nt	nt	+	+	+	+	+	+	+	+	+
	2036-1			+	+	+	+	nt	nt	+	+	+	+	+	+	+	+	+
	2133			+	+	+	+	nt	nt	+	+	+	+	+	+	+	+	+
	2134-2			+	+	+	+	nt	nt	+	+	+	+	+	+	+	+	+
	2134-3			+	+	+	+	nt	nt	+	+	+	+	+	+	+	+	+
	2072	Opatów, świętokrzyskie, PL	2020	+	+	+	+	nt	nt	+	+	+	+	+	+	+	+	+
	2076-1			+	+	+	+	nt	nt	+	+	+	+	+	+	+	+	+
	2077-1			+	+	+	+	nt	nt	+	+	+	+	+	+	+	+	+
	2078			+	+	+	+	nt	nt	+	+	+	+	+	+	+	+	+

JL2600	Oregon, USA (cv. 'Dorris')		+	?[3]	?[4]	+	+	+	+	+	+	+	+	+	+	+	+
JL2602	Oregon, USA (cv. 'McDonald')		+	+	+	+	+	+	nt	nt	nt	nt	nt	nt	nt	nt	nt
JL2606			+	+	+	+	+	+	+	+	+	+	+	+	+	+	+
JL2607	Oregon, USA (cv. 'Ennis')		+	+	+	+	+	+	+	+	+	+	+	+	+	+	+
JL2603			+	+	+	+	+	+	+	+	+	+	+	+	+	+	+
JL2605	Oregon, USA (cv. 'Wepster')	2017	+	+	+	+	+	+	nt	nt	nt	nt	nt	nt	nt	nt	nt
JL2610			+	+	+	+	+	+	+	+	+	+	+	+	+	+	+
JL2611			+	+	+	+	+	+	nt	nt	nt	nt	nt	nt	nt	nt	nt
JL2612			+	+	+	+	+	+	+	+	+	+	+	+	+	+	+
JL2613	Oregon, USA (cv. 'Jefferson')		+	+	+	+	+	+	nt	nt	nt	nt	nt	nt	nt	nt	nt
JL2614			+	+	+	+	+	+	nt	nt	nt	nt	nt	nt	nt	nt	nt
JL2615			+	+	+	+	+	+	nt	nt	nt	nt	nt	nt	nt	nt	nt
JL2616	Oregon, USA (cv. 'Jefferson')		+	+	+	+	+	+	+	+	+	+	+	+	+	+	+
JL2617		2018	+	+	+	+	+	+	nt	nt	nt	nt	nt	nt	nt	nt	nt
JL2618			+	+	+	+	+	+	+	+	+	+	+	+	+	+	+
LfL 05/113/2a	DE	2005	+	+	?[5]	+	+	+	+	+	+	+	nt	nt	+	+	+
LfL 05/139/2a			+	+	+	+	+	+	+	+	+	+	nt	nt	+	+	+
LfL 09/162/1a		2009	+	+	+	+	+	+	+	+	+	+	nt	nt	+	+	+
LfL 07/39/1a		2007	+	+	+	+	+	+	+	+	+	+	nt	nt	+	+	+
LfL 06/102/3a		2006	+	+	+	+	+	+	+	+	+	+	nt	nt	+	+	+
KFB275	Petrovčić, RS	2008	nt	nt	nt	nt	+	+	nt	nt	nt	nt	+	nt	nt	nt	nt
KFB 282		2009	nt	nt	nt	nt	+	+	nt	nt	nt	nt	+	nt	nt	nt	nt
KFB 288	Deč, RS	2010	nt	nt	nt	nt	+	+	nt	nt	nt	nt	+	nt	nt	nt	nt
KFB 289		2010	nt	nt	nt	nt	+	+	nt	nt	nt	nt	+	nt	nt	nt	nt
KFB 308	Erdevik, RS	2010	nt	nt	nt	nt	+	+	nt	nt	nt	nt	+	nt	nt	nt	nt
KFB 314	Požarevac, RS	2011	nt	nt	nt	nt	+	+	nt	nt	nt	nt	+	nt	nt	nt	nt
RKFB 822	plant material import from IT	2014	nt	nt	nt	nt	+	+	nt	nt	nt	nt	+	nt	nt	nt	nt
RKFB 829		2014	nt	nt	nt	nt	+	+	nt	nt	nt	nt	+	nt	nt	nt	nt
RKFB 835		2015	nt	nt	nt	nt	+	+	nt	nt	nt	nt	+	nt	nt	nt	nt
RKFB 1084	Jakovo, RS	2016	nt	nt	nt	nt	+	+	nt	nt	nt	nt	+	nt	nt	nt	nt
RKFB 1110	plant material import from HU	2016	nt	nt	nt	nt	+	+	nt	nt	nt	nt	+	nt	nt	nt	nt
RKFB 1227	plant material import from DE	2019	nt	nt	nt	nt	+	+	nt	nt	nt	nt	+	nt	nt	nt	nt
NCPPB 3037	UK	1977	nt	nt	nt	nt	+	+	nt	nt	nt	nt	+	nt	nt	nt	nt
NCPPB 935[PT]	Oregon, USA	1939	nt	nt	nt	nt	+	+	nt	nt	nt	nt	+	nt	nt	nt	nt
KFB0125, XA 3.75	DE	1999	nt	nt	nt	nt	+	+	nt	nt	nt	nt	+	nt	nt	nt	nt

	KFB0126, NCPPB 3339; XA 5.25	FR	1984	nt	nt	nt	nt	+	+	nt	nt	nt	nt	+	nt	nt	nt	nt
	KFB0134, RIPF X18	PL	2009	nt	nt	nt	nt	+	+	nt	nt	nt	nt	+	nt	nt	nt	nt
X. arboricola pv. arracaciae	CFBP 7407^{PT}	BR	1969	-	-	-	-	nt	nt	-	-	-	-	-	-	-	-	-
X. arboricola pv. zantedeschiae	CFBP 7410^{PT}	SA	1967	-	-	-	-	nt	nt	-	-	-	-	-	-	-	-	-
X. arboricola pv. celebensis	CFBP 3523 ^{PT}	NZ	1960	-	-	-	-	nt	nt	-	-	-	-	-	-	-	-	-
	NCPPB 1832		1966	nt	nt	nt	nt	-	-	nt	-	nt	nt	nt	nt	nt	nt	nt
X. arboricola pv. fragariae	CFBP 6771 ^{PT}	Cesena, IT	2001	-	-	-	-	nt	nt	-	-	-	-	-	-	-	-	-
	NCPPB 4182	IT	2000	nt	nt	nt	nt	-	-	nt	nt	nt	nt	nt	nt	nt	nt	nt
	CFBP 2535^{PT}	Auckland Mt Albert, NZ	1953	-	-	-	-	nt	nt	-	-	-	-	-	-	-	-	-
X. arboricola pv. pruni	KFB0146	NL	1953	nt	nt	nt	nt	-	-	nt	nt	nt	nt	nt	nt	nt	nt	nt
	KFB0152	BE	2009	nt	nt	nt	nt	-	-	nt	nt	nt	nt	nt	nt	nt	nt	nt
	KFB0104, 69VR	IT	1992	nt	nt	nt	nt	-	-	nt	nt	nt	nt	nt	nt	nt	nt	nt
	CFBP 2528^{T}	NZ	1956	-	-	-	-	-	-	-	-	-	-	-	-	-	-	-
	CFBP 7179	FR	2002	-	-	-	-	nt	nt	-	-	-	-	-	-	-	-	-
	I-391	PT	1994	-	-	-	-	nt	nt	-	-	-	-	-	-	-	-	-
	LMG 746	UK	1955	-	-	-	-	nt	nt	-	-	-	-	-	-	-	-	-
	506	Ostrzeszów, southwest region, PL	2008	-	-	-	-	nt	nt	-	-	-	-	-	-	-	-	-
	507			-	-	-	-	nt	nt	-	-	-	-	-	-	-	-	-
	508			-	-	-	-	nt	nt	-	-	-	-	-	-	-	-	-
X. arboricola pv. juglandis	509			-	-	-	-	nt	nt	-	-	-	-	-	-	-	-	-
	510			-	-	-	-	nt	nt	-	-	-	-	-	-	-	-	-
	539	Zawady near Częstochowa, south region, PL	2008	-	-	-	-	nt	nt	-	-	-	-	-	-	-	-	-
	540			-	-	-	-	nt	nt	-	-	-	-	-	-	-	-	-
	541			-	-	-	-	nt	nt	-	-	-	-	-	-	-	-	-
	2029-2	Zalesie, łódzkie, PL	2020	-	-	-	-	nt	nt	-	-	-	-	nt	nt	-	-	-
	2030-1			-	-	-	-	nt	nt	-	-	-	-	nt	nt	-	-	-
	2087-1	Skierniewice, łódzkie, PL	2020	-	-	-	-	nt	nt	-	-	-	-	nt	nt	-	-	-
	2089			-	-	-	-	nt	nt	-	-	-	-	nt	nt	-	-	-
	2099	Jantar, pomorskie, PL	2020	-	-	-	-	nt	nt	-	-	-	-	nt	nt	-	-	-
Xanthomonas guizotiae	CFBP 7408^{PT}	ET	1964	-	-	-	-	nt	nt	-	-	-	-	-	-	-	-	-
Xanthomonas populina	CFBP 3123 ^{PT}	NL	1979	-	-	-	-	nt	nt	-	-	-	-	-	-	-	-	-
	NCPPB 2987	UK	1977	nt	nt	nt	nt	-	-	nt	nt	nt	nt	nt	nt	nt	nt	nt

Pseudomonas avellanae	CFBP 4060	GR	1976	-	-	-	-	nt	nt	-	-	-	-	-	-	-	-	-
	2005-4	Skierniewice, łódzkie, PL	2020	-	-	-	-	nt	nt	-	-	-	-	-	-	-	-	-
	727	Skierniewice, łódzkie, PL	1997	-	-	-	-	nt	nt	-	-	-	-	-	-	-	-	-
	749		2000	-	-	-	-	nt	nt	-	-	-	-	nt	nt	-	-	-
	1811-2	Wrocław, dolnośląskie, PL	2019	-	-	-	-	nt	nt	-	-	-	-	nt	nt			
HR positive *Pseudomonas* isolated from hazelnut	2005-2	Skierniewice, łódzkie, PL	2020	-	-	-	-	nt	nt	-	-	-	-	nt	nt	-	-	-
	2049			-	-	-	-	nt	nt	-	-	-	-	nt	nt			
	2052 C	Motycz, Lubelskie, PL	2020	-	-	-	-	nt	nt	-	-	-	-	nt	nt	-	-	-
	2055-3			-	-	-	-	nt	nt	-	-	-	-	nt	nt	-	-	-
	2069	Opatów, świętokrzyskie, PL	2020	-	-	-	-	nt	nt	-	-	-	-	nt	nt			
	2101	Jantar, pomorskie, PL	2020	-	-	-	-	nt	nt	-	-	-	-	-	-	-	-	-
Sphingomonas sp. (non-pathogenic on hazelnut)	JL2604	Oregon, USA	2017	-	-	-	-	-	-	nt	nt	nt	nt	nt	nt	nt	nt	nt
Xanthomonas campestris (non-pathogenic on hazelnut)	JL2609	Oregon, USA	2017	-	-	-	-	-	-	nt	nt	nt	nt	nt	nt	nt	nt	nt
HR positive *Pseudomonas* isolated from walnut	2001-5	Skierniewice, łódzkie PL	2020	-	-	-	-	nt	nt	-	-	-	-	nt	nt	nt	nt	nt
	2002-3			-	-	-	-	nt	nt	-	-	-	-	nt	nt	-	-	-
	2098-1 Ś	Jantar, pomorskie, PL	2020	-	-	-	-	nt	nt	-	-	-	-	nt	nt	-	-	-
	298	Pamiętna, łódzkie, PL	2007	nt	nt	nt	nt	nt	nt	-	-	-	-	nt	nt	-	-	-
HR negative *Pseudomonas* and other isolates obtained from hazelnut	840	Skierniewice, łódzkie, PL	1998	-	-	-	-	nt	nt	-	-	-	-	nt	nt	-	-	-
	2038	Motycz, Lubelskie, PL	2020	-	-	-	-	nt	nt	-	-	-	-	nt	nt	-	-	-
	2045B			-	-	-	-	nt	nt	-	-	-	-	nt	nt	-	-	-
	2053 D			-	-	-	-	nt	nt	-	-	-	-	nt	nt	-	-	-
	2061A			-	-	-	-	nt	nt	-	-	-	-	nt	nt	-	-	-
	2068B1	Opatów, świętokrzyskie, PL	2020	-	-	-	-	nt	nt	-	-	-	-	nt	nt	-	-	-
	2136	Skierniewice, łódzkie PL	2020	-	-	-	-	nt	nt	-	-	-	-	nt	nt	-	-	-
HR negative *Pseudomonas*	514		2008	-	-	-	-	nt	nt	-	-	-	-	nt	nt	-	-	-
	521	Bełchów, łódzkie, PL	2008	-	-	-	-	nt	nt	-	-	-	-	nt	nt	-	-	-

and other isolates obtained from walnut	531	Karolew, łódzkie, PL	2008	-	-	-	-	nt	nt	-	-	-	-	nt	nt	-	-	-
	533	Bednary, łódzkie, PL	2008	-	-	-	-	nt	nt	-	-	-	-	nt	nt	-	-	-
	575	Aleksandrów Łódzki, PL	2008	-	-	-	-	nt	nt	-	-	-	-	nt	nt	-	-	-
	1813-2	Wrocław, dolnośląskie, PL	2020	-	-	-	-	nt	nt	-	-	-	-	nt	nt	-	-	-
	2030-1 Ś	Zalesie, łódzkie, PL	2020	-	-	-	-	nt	nt	-	-	-	-	nt	nt	-	-	-
	2099	Jantar, pomorskie, PL	2020	-	-	-	-	nt	nt	-	-	-	-	nt	nt	-	-	-
DNA from healthy plants (GeneMATRIX Plant & Fungi DNA Purification Kit)	A/Cosford			-	-	-	-	nt	nt	-	-	-	-	nt	nt	-	-	-
	B/Cud z Boliwier			-	-	-	-	nt	nt	-	-	-	-	nt	nt	-	-	-
	C/Carribaldi			-	-	-	-	nt	nt	-	-	-	-	nt	nt	-	-	-
	D/Webba	Nursery	2020	-	-	-	-	nt	nt	-	-	-	-	nt	nt	-	-	-
	E/Olbrzymi z Halle			-	-	-	-	nt	nt	-	-	-	-	nt	nt	-	-	-
Fungi isolated from diseased hazelnut	*Didymella* sp 2136A 2136B	Skierniewice, łódzkie, PL	2020	-	-	-	-	nt	nt	-	-	-	-	nt	nt	nt	nt	nt
	Peyronellae sp. 2005			-	-	-	-	nt	nt	-	-	-	-	nt	nt	nt	nt	nt
	Fusarium sp. 2068 B	Opatów, świętokrzyskie, PL	2020	-	-	-	-	nt	nt	-	-	-	-	nt	nt	nt	nt	nt
	Botrytis cinerea 2066			-	-	-	-	nt	nt	-	-	-	-	nt	nt	nt	nt	nt
	Alternaria sp. 2109-1	Dębowa Góra, łódzkie, PL	2020	-	-	-	-	nt	nt	-	-	-	-	nt	nt	nt	nt	nt
	Alternaria sp. 2109-2			-	-	-	-	nt	nt	-	-	-	-	nt	nt	nt	nt	nt
Fungi isolated from diseased walnut	*Colletotrichum* sp. OW A			-	-	-	-	nt	nt	-	-	-	-	nt	nt	nt	nt	nt
	Diaporthe sp. OW C			-	-	-	-	nt	nt	-	-	-	-	nt	nt	nt	nt	nt
	Didymellaceae sp. OW B, OW D	Skierniewice, łódzkie PL	2020	-	-	-	-	nt	nt	-	-	-	-	nt	nt	nt	nt	nt

[1]The culture collections providing the strain is abbreviated in the strain name as CFBP (Collection Française de Bactéries Associées aux Plantes, Beaucouzé, France), LMG, NCPPB.
[2]The first four columns for qPCR highlighted in light blue correspond to assays performed with SYBR Green I whereas the fifth and sixth column highlighted in grey correspond to TaqMan assays.
[3]A 900 bp amplicon was observed instead of the 1’455 bp expected amplicon.
[4]A 1,450 bp amplicon was observed instead of the 385 bp expected amplicon.
[5]A 1,100 bp amplicon was observed instead of the 385 bp expected amplicon.
(+): amplification observed with expected size; (-): no amplification observed; nt: not tested.
